# Patient Characteristics in Hemodialysis Mortality: A Comparison of Cerebro-Cardiovascular and Renal Failure Deaths in a Regional Core Hospital in Japan

**DOI:** 10.7759/cureus.91074

**Published:** 2025-08-26

**Authors:** Taku Goto, Takuya Kishi, Shiki Nakayama, Koji Onozawa, Kohei Yamanouchi, Akira Kitajima, Kazuma Fujimoto, Ayako Takamori, Yuichiro Sakamoto

**Affiliations:** 1 Emergency Medicine, Department of Graduate School of Medicine, International University of Health and Welfare, Okawa, JPN; 2 Emergency, Kouhou-kai Takagi Hospital, Okawa, JPN; 3 Cardiology, Department of Graduate School of Medicine, International University of Health and Welfare, Okawa, JPN; 4 Nephrology, Kouhou-kai Takagi Hospital, Okawa, JPN; 5 Gastroenterology, Kouhou-kai Takagi Hospital, Okawa, JPN; 6 Gastroenterology, Department of Graduate School of Medicine, International University of Health and Welfare, Okawa, JPN; 7 Clinical Research Center in Hiroshima, Hiroshima University Hospital, Hiroshima, JPN; 8 Faculty of Medicine, Department of Emergency Medicine, Saga University, Saga, JPN

**Keywords:** age, body mass index, cardiovascular events, cerebrovascular events, hemodialysis

## Abstract

Patients with renal failure undergoing hemodialysis have a reduced survival rate. Patients with hemodialysis are well known to frequently experience complications such as infections, cerebro-cardiovascular disorders, and gastrointestinal bleeding. At the central hospital where we work in a rural area of Japan, we treat patients with hemodialysis until death, including cases with sudden changes in condition, and all clinical data leading to death are collected. Additionally, compared to previous reports on patients with hemodialysis, our hospital has a higher proportion of elderly patients, and deaths due to cerebro-cardiovascular diseases are also not uncommon. Overseas reports on whether there are differences in the underlying factors between deaths due to cerebro-cardiovascular disease and those due to renal failure in patients with hemodialysis have shown that obesity has a protective effect against deaths due to cerebro-cardiovascular disease, but the protective effect of obesity is limited in deaths due to renal failure. However, there are no reports on this point in Japan.

To address this knowledge gap, the primary objective of this study was to compare patient characteristics between groups of patients with hemodialysis at our regional core hospital who died from cerebro-cardiovascular disease and those who died from renal failure. Continuous variables were assessed for normality using the Shapiro-Wilk test and visual inspection of histograms and Q-Q plots. Normally distributed continuous variables were compared using Student's t-test for two groups or one-way analysis of variance for multiple groups. Non-normally distributed continuous variables were analyzed using the Mann-Whitney U test for two groups or the Kruskal-Wallis test for multiple groups. Categorical variables were compared using the Chi-square test or Fisher's exact test. A total of 151 patients diagnosed with end-stage renal disease who were undergoing hemodialysis at the dialysis center of the nephrology department of our hospital as of December 2017 and had no history of cerebro-cardiovascular disease were enrolled in the study. All patients were followed up for five years or until death, whichever occurred first, until December 2022, and continuous monitoring of all diseases was conducted. The annual mortality rate among patients receiving dialysis was relatively high at 7.3%. Compared with the survival group, the mortality group had a higher proportion of males, older age, and bone fractures, as well as lower serum iron levels. Patients who died from cerebro-cardiovascular disease were significantly younger and had higher systolic blood pressure and higher body mass index (BMI) than those who died from renal failure. Additionally, among patients with a BMI of 22 or higher, deaths from cerebro-cardiovascular disease were significantly more common than those from renal failure.

In conclusion, among patients with hemodialysis at regional core hospitals in Japan, those who died from cerebro-cardiovascular disease were significantly younger and had higher systolic blood pressure and BMI compared to those who died from renal failure. Although this finding differs from previous reports, it suggests that in the management of patients with hemodialysis at rural core hospitals in Japan, where the elderly population is high, it may be necessary to actively manage blood pressure and atherosclerosis while maintaining a BMI below 22 in younger patients.

## Introduction

The central therapeutic approach for end-stage renal failure patients is maintenance hemodialysis, and there are several risk factors during the therapeutic period, including background patient characteristics and conditions, comorbidities, and adverse events [1-3]. Several reports indicate that patients with renal failure treated by hemodialysis have low rates of life expectancy due to life-threatening factors such as adverse events and complications [4-6]. It is well known that infection, cerebro-cardiovascular disorders, and gastrointestinal bleeding are often complicated in patients treated by maintenance hemodialysis due to hemodialysis itself and the original diseases of end-stage renal failure [4-6]. Several diseases, including cerebro-cardiovascular disorders and gastrointestinal bleeding, are reported to worsen the life prognosis of hemodialysis patients [3,7,8].

In Japan, hemodialysis for end-stage renal failure is guaranteed by a universal insurance system that is nearly free for patients regardless of age, performance status, comorbidities, and underlying diseases causing renal failure [9]. However, the wide range of therapeutic indications may increase the risks of adverse events and mortality during hemodialysis, and several studies suggest several risk factors and leading causes of mortality [1-3]. At the central hospital in the rural area of Japan where we work, we treat patients with hemodialysis until their death, and we often have data on the clinical course leading up to death. Previously, we have reported that, in addition to advanced age and low body weight, fractures of the lower extremities are important risk factors for mortality in dialysis patients [8,10]. Furthermore, compared to previous reports, our hospital has a higher proportion of elderly patients [11-13]. In patients with hemodialysis, overseas reports indicate that obesity has a protective effect against deaths from cerebro-cardiovascular diseases [14-16]. In patients with hemodialysis who died from renal failure, low body mass index (BMI) was also associated with an increased risk of hemodialysis discontinuation [17]. On the other hand, a protective effect of high BMI was observed in inflamed patients, and this effect was mitigated in noninflamed patients [16]. Furthermore, a previous report suggested that there were no positive associations of malnutrition with documented acute coronary syndromes requiring hospitalization in a large incident Medicare dialysis population [18]. Unlike reports from overseas, in Japan, high BMI may increase the risk of death in hemodialysis patients in the long term [19]. Considering these backgrounds, if there is no excessive inflammation, a high BMI may increase the risk of death in Japanese patients with hemodialysis, particularly from cerebro-cardiovascular disease, compared to renal failure. However, there are no reports from Japan on this point.

To fill this knowledge gap, the primary objective of the current study was to compare patient characteristics among the mortality groups with cerebro-cardiovascular diseases and those with renal failure within the maintenance hemodialysis patient population at our regional core hospital in Japan. Since this study is a retrospective study conducted at a single institution, selection bias cannot be avoided, and the results cannot be generalized. The limitations of this study include the fact that the results merely describe the current situation at a single institution in Japan. We acknowledge that the findings of this study may not be representative or statistically significant enough to be generalized to a broader population. However, this study was initiated with the aim of understanding and analyzing the current situation at core hospitals in rural areas of Japan, to inform future clinical decision-making.

## Materials and methods

This study was a single-center, retrospective, observational study. The inclusion criteria are as follows: patients with end-stage renal failure undergoing maintenance dialysis at the dialysis center of the nephrology division of Takagi Hospital (a regional core hospital) in December 2017. The exclusion criteria are as follows: 1) patients who have been switched to treatment at a medical institution other than Takagi Hospital, 2) patients with a history of cerebro-cardiovascular disease, including coronary artery disease or peripheral artery disease, were excluded from this study because they could significantly influence the results. Finally, 151 patients were included in the study. All patients were followed up until December 2022, either for five years or until death, and their diseases were monitored continuously at Takagi Hospital without dropout. Data on patient characteristics and event incidences, including death, were collected in the electronic medical record over a five-year period until December 2022. Regarding the completeness and accuracy of electronic medical records, the patients included in this study were selected from a dialysis patient database used by the attending physicians in their daily practice, and the data were anonymized and analyzed by researchers who were not the attending physicians.

We extracted data from a hemodialysis patient database used in routine clinical practice at dialysis centers, anonymized it, and conducted the analysis by researchers who were not the attending physicians. Gender, age, body mass index, smoking habits, duration of dialysis, origin of renal failure, blood pressure, hemoglobin, serum total protein, serum total cholesterol, serum calcium, serum parathyroid hormone (transformed using the natural logarithm), and iron concentration were compared in mortality and survival groups in December 2017. We calculated BMI using post-dialysis weight. Blood tests were done before meals and hemodialysis. Blood pressure was measured before dialysis, and the average of the three measurements taken during the same week was used as the baseline value. This measurement was taken during the period after dialysis was initiated, when blood pressure had stabilized and dialysis could be performed three times a week. Intact parathyroid hormone was evaluated using log-transformed data because concentrations were skewed to the left. There was no missing data for the items analyzed in the databases used. Incidence of bone fracture and gastrointestinal bleeding during the follow-up period was detected. Regarding the cause of death, all patients who died during the study period were confirmed dead either during hospitalization at our hospital or as outpatients, and multiple physicians, including the attending physician at the dialysis center, determined the cause of death and recorded it in the medical records and database.

In this study, based on previous reports, we defined the cause of death as either death due to uremic symptoms following dialysis cessation caused by vascular access failure or death due to toxin accumulation caused by inadequate dialysis efficiency [20]. A total of 55 mortality patients during the five-year follow-up period were classified into five groups according to the cause of death: renal failure (n=18), infection (n=16), cerebro-cardiovascular diseases (n=12), others (n=7), and malignancy (n=2). Among 12 patients who died from cerebro-cardiovascular diseases, five had acute myocardial infarction, two had cerebral hemorrhage, two had cerebral infarction, one had aortic dissection, and one had lower extremity acute arterial occlusion. A patient died of aortic dissection at home, and other patients died in Takagi Hospital. The characteristics of patients in each group were compared to those of the group of patients who died of renal failure. The study was conducted in accordance with the Declaration of Helsinki, with ethical review and approval from Takagi Hospital's Ethical Committee (Kouhou-kai Ethical Committee: KR215) and the International University of Health and Welfare Ethical Committee (23-Ifh-036). Informed consent was obtained from the survivors and the families of the deceased under the guidelines of the Declaration of Helsinki.

Continuous variables were assessed for normality using the Shapiro-Wilk test and visual inspection of histograms and Q-Q plots. Normally distributed continuous variables were compared using Student's t-test for two groups or one-way analysis of variance for multiple groups, and presented as means ± standard deviations. Non-normally distributed continuous variables were analyzed using the Mann-Whitney U test for two groups or the Kruskal-Wallis test for multiple groups, and presented as medians with interquartile ranges. Categorical variables were compared using the Chi-square test or Fisher's exact test when expected cell counts were less than five. Results were presented as frequencies and percentages. JMP Pro 16 software (SAS Institute Inc., Cary, NC, USA) was used for all analyses, and statistical significance was defined as p < 0.05.

## Results

Table 1 compares mortality and survival of patients treated with hemodialysis. During the five-year follow-up period from December 2017 to December 2022, 55 patients (36.4%) out of 151 died during hemodialysis, and 7.3% of the hemodialysis patients, on average, died per year. The mortality rate was significantly higher in males than in females (p=0.03), and the age of the mortality group was significantly older than that of the survival group (p<0.01). Lower body fracture was more complicated in the mortality group compared to the survival group (p=0.013). There was no difference between the two groups in body mass index, duration of hemodialysis, origin of renal failure, upper body bone fracture, and gastrointestinal bleeding. 

**Table 1:** The characteristics of the mortality and survival patients treated with hemodialysis during December 2017 and December 2022 * =p<0.05, and ** =p<0.01. Data are mean ± SD. Nephrogenic diseases, including chronic nephritis and nephrosclerosis. Continuous variables were assessed for normality using the Shapiro-Wilk test and visual inspection of histograms and Q-Q plots. Normally distributed continuous variables were compared using Student's t-test for two groups and presented as means ± standard deviations. Non-normally distributed continuous variables were analyzed using the Mann-Whitney U test for two groups and presented as medians with interquartile ranges. Categorical variables were compared using the chi-square test or Fisher's exact test when expected cell counts were less than 5. Results were presented as frequencies and percentages.

	Death	Survival	P value
Numbers	55	96	
Males	39 (71%)	51 (53%)	0.03*
Age (year)	75.0±11.4	65.2±12.6	<0.01**
Body mass index (kg/m^2^)	21.5±5.2	21.3±3.6	0.79
Duration of hemodialysis (month)	110.4±118.9	103.0±94.8	0.67
Origin of renal failure			
Diabetes mellitus	18 (33%)	36 (38%)	0.18
Nephrogenic diseases	37 (67%)	60 (63%)	0.18
Bone fracture			
Upper body	4 (7%)	8 (8%)	0.82
Lower body	15 (27%)	11 (11%)	0.013*
Gastrointestinal bleeding	17 (31%)	19 (20%)	0.08

Table 2 presents the blood test results of the patients with hemosialysis at the time of their inclusion in the present study in December 2017. The serum iron level was significantly higher in the survival group than in the mortality group (p=0.011), and other factors, including hemoglobin, total protein, cholesterol, serum calcium, and intact parathyroid hormone (evaluated using log-transformed data), were not different between the mortality and survival groups. 

**Table 2:** Blood test of the patients with hemodialysis in December 2017: comparison between the mortality patients and the survival patients with hemodialysis iPTH; intact parathyroid hormone *p<0.05. data are mean± SD. iPTH was evaluated using log-transformed data because concentrations were skewed to the left.

	Mortality	Survival	P value
Number	55	96	
Hemoglobin (g/dL)	10.9±1.7	10.9±1.4	0.84
Total protein (g/dL)	6.3±0.6	6.3±0.6	0.49
Cholesterol (mg/dL)	141±43	151±35	0.15
Corrected serum calcium (mg/dL)	8.9±0.7	9.0±0.6	0.29
ln (iPTH (pg/mL))	4.87±0.93	4.76±0.78	0.23
Serum iron (mg/dL)	54±25	65±27	0.011*

Table 3 shows the causes of mortality of hemodialysis patients. As indicated, 18 patients (32.7%) died due to renal failure, and 16 patients died because of infection. The number of patients who died from cerebro-cardiovascular diseases was 12, which was equivalent to the number of patients with renal failure and infection. Seven patients died due to malignancy. Almost all the patients died in Takagi Hospital, and two patients died at their homes (unknown cause of mortality). Compared to the patients who died because of renal failure, the age of the patients who died with cerebro-cardiovascular diseases was significantly younger (p<0.01). Compared to the patients who died of renal failure, the BMI of patients who died of cerebro-cardiovascular diseases was significantly higher (p<0.05). In the group with a BMI of 22 or higher, the number of deaths from cerebro-cardiovascular disease was significantly higher than that from renal failure. Systolic blood pressure was significantly higher in the group with death from cerebro-cardiovascular disease than in the group with death from renal failure. There was no significant difference in diastolic blood pressure between the two groups. There was no patient wth gastrointestinal bleeding in the patients who died from cerebro-cardiovascular diseases. Other factors, including gender, smoking habits, hemodialysis duration, origin of renal failure, and complications during hemodialysis, were not different among the groups.

**Table 3:** The causes of the mortality classified the mortality patients during the hemodialysis during a five-year follow-up period Then causes were renal failure, infectious diseases, cerebro-cardiovascular diseases, malignancy, and unknown. CCD; cerebro-cardiovascular diseases, BMI; body mass index, BP; blood pressure, HD; hemodialysis * p<0.05 and ** p<0.01, compared to the renal failure group. Continuous variables were assessed for normality using the Shapiro-Wilk test and visual inspection of histograms and Q-Q plots. Normally distributed continuous variables were compared using Student's t-test for two groups or one-way analysis of variance (ANOVA) for multiple groups, and presented as means ± standard deviations. Non-normally distributed continuous variables were analyzed using the Mann-Whitney U test for two groups or the Kruskal-Wallis test for multiple groups and presented as medians with interquartile ranges. Categorical variables were compared using the Chi-square test or Fisher's exact test when expected cell counts were less than 5. Results were presented as frequencies and percentages.

	Renal failure	Infection	CCD	Malignance	Unknown
Number	18	16	12	7	2
Males / Females	13 / 5	11 / 5	8 / 4	6 / 1	1 / 1
Age (year)	77 ± 9	76 ± 12	65 ± 11**	82 ± 7	81 ± 9
BMI (kg/m^2^)	19.7 ± 3.3	20.5 ± 3.5	25.1 ± 8.6*	22.5 ± 1.9*	19.8 ± 1.1
BMI≧22	6 (33%)	4 (25%)	8 (67%)**	3 (43%)	0 (0%)
Smoking	1 (6%)	1 (13%)	1 (8%)	1 (14%)	0 (0%)
Systolic BP (mmHg)	131 ± 3	130 ± 6	138 ± 6*	132 ± 2	131 ± 2
Diastolic BP (mmHg)	79 ± 4	75 ± 4	78 ± 5	71± 6	75± 1
HD duration (month)	110 ± 124	152 ± 145	71 ± 48	106 ± 131	39 ± 31
Origin of renal failure					
Diabetes mellitus	7 (39%)	3 (19%)	6 (50%)	2 (29%)	1 (50%)
Nephrogenic diseases	12 (67%)	13 (81%)	7 (58%)	5 (71%)	1 (50%)
Lower body bone fracture	6 (33%)	4 (25%)	4 (33%)	2 (29%)	1 (50%)
Gastrointestinal bleeding	6 (33%)	6 (38%)	0 (0%)*	3 (43%)	2 (100%)

## Discussion

The five-year follow-up study indicated that (i) The mortality rate of patients with hemodialysis was relatively high, 7.3% per year; (ii) The group that died had significantly more patients with lower limb fractures and significantly lower serum iron levels (iii) Patients who died from cerebro-cardiovascular diseases were significantly younger, with higher BMI and systolic blood pressure, compared to patients who died from renal failure.

The mortality rate of hemodialysis was relatively high, and the deceased group was significantly older than the survivors and had more males. Gender differences in life expectancy among hemodialysis patients are almost identical, with women reported to have a significantly worse prognosis in patients with coexisting cardiovascular disease, including coronary artery disease [21]. In Japan, however, it has been reported that the proportion of men in cardiovascular disease-related deaths is higher in younger patients, and the gender difference disappears in older patients [19]. Furthermore, a cohort study of newly inducted dialysis patients reported significantly higher rates of pre-existing and comorbid diabetes and cardiovascular disease in men. Therefore, the results of the present study do not differ from previous reports.

Lower-body bone fractures might be a risk for mortality. Our previous studies have also shown that the prognosis of dialysis patients is significantly worse when associated with lower limb fractures [10]. The mechanism remains speculative, but previous studies in Japan have suggested that frailty is a risk factor for survival outcomes in hemodialysis patients [22,23]. We speculate that the time constraints on physical rehabilitation following lower limb fractures in hemodialysis patients may hinder smooth recovery from fractures and lead to mobility restrictions caused by lower limb fractures. Lower limb fractures reduce activities of daily living and lead to a deterioration in nutritional status, which may be a factor in worsening life expectancy. To clarify this point, future studies that examine nutritional status and bone metabolism-related markers in detail will be helpful.

In this study, it is noteworthy that patients who died from cerebro-cardiovascular disease were significantly younger than those who died from renal failure. Although patients with cerebro-cardiovascular disease at the time of hemodialysis initiation were excluded from this study, previous reports have indicated that approximately half of patients had coronary artery stenosis at the start of dialysis [24]. On the other hand, a study of 2,966 Japanese patients with chronic kidney disease, followed for more than four years, found that the incidence of new cardiovascular disease in Japanese patients with chronic kidney disease was lower than in studies conducted in the United States [25,26]. The incidence of cerebro-cardiovascular disease in Japan may be lower than in other countries because patients with chronic kidney disease are under the care of nephrologists, and anemia, blood pressure, and blood sugar are appropriately managed. However, in this study, the group that died from cerebro-cardiovascular disease had significantly higher systolic blood pressure compared to the group that died from kidney failure. Therefore, even in younger patients undergoing hemodialysis, managing atherosclerosis risk, particularly hypertension, may help prevent death from cerebro-cardiovascular disease.

We found that patients who died from cerebro-cardiovascular diseases had significantly higher BMI compared to patients who died from renal failure, and that in the BMI of 22 or higher group, the cerebro-cardiovascular diseases group had significantly more deaths compared to the renal failure group. Several overseas studies have suggested that obesity has a protective effect against deaths from cerebro-cardiovascular diseases [14-16]. However, unless there is excessive inflammation, a high BMI may increase the risk of death in Japanese patients with hemodialysis, particularly from cerebrovascular disease. The results of this study suggest that in patients with a BMI of 22 or higher, death from cerebro-cardiovascular disease was significantly more common than death from renal failure, and may have identified characteristics of Japanese hemodialysis patients that are not known from overseas reports. While we cannot conclude from the results of this study, we considered obesity-related atherosclerosis as the potential reason for the association between high BMI and death from cerebro-cardiovascular disease. In addition, to prevent deaths from cerebro-cardiovascular disease among dialysis patients at core hospitals in rural areas of Japan, it may be essential to manage lifestyle habits to maintain a BMI of around 22, in addition to the aforementioned blood pressure management.

Regarding the significantly lower serum iron concentration in the mortality group, we also considered potential mechanisms such as chronic inflammation and malnutrition [27]. It is difficult to draw a conclusion because we did not obtain sufficient data to evaluate nutritional status adequately. However, although not statistically significant, the tendency toward lower cholesterol levels in the mortality group suggests a possible association. Regarding chronic inflammation, there is insufficient data to conduct a comprehensive investigation. This has been identified as a future area for consideration.

No cases of gastrointestinal bleeding were observed in the group of deaths due to cerebro-cardiovascular disease. While previous studies and our studies have shown that gastrointestinal bleeding is common in dialysis patients [8-13], the small sample size in this study makes it difficult to interpret the results. Additionally, five patients in the group with deaths due to cerebro-cardiovascular disease were taking anticoagulants or antiplatelet agents for conditions such as atrial fibrillation or coronary artery disease; however, the number of cases was too small to analyze the impact of these medications. We consider that this may be due to sample size limitations, not a protective factor, and that anticoagulant or antiplatelet usage di not influence this result.

The limitations of the present retrospective follow-up study were the relatively small sample size and the five-year follow-up period of patients undergoing maintenance hemodialysis. This study is a retrospective analysis conducted at a single institution; selection bias cannot be avoided, and the results cannot be generalized, but merely describe the current situation at a single institution in Japan. Furthermore, due to insufficient sample size and data collection, it was not possible to perform accurate multivariate analysis. Therefore, it was not possible to examine causal relationships based on the results of this study. We did not analyze the influence of medication use, socioeconomic status, or physical activity, nor the influence of regional differences (diet, medical system, or genetic factors) on mortality and outcomes in patients with hemodialysis. As a result, this study was limited to describing differences in clinical backgrounds. However, it is essential to note that all 151 patients were closely monitored at Takagi Hospital and received comprehensive medical care, and the cause of death was confirmed at Takagi Hospital, except for two patients who died in their homes.

## Conclusions

Our five-year follow-up retrospective and small study conducted at a single institution has the potential to significantly impact patient care. We found that patients with hemodialysis who succumbed to cerebro-cardiovascular diseases were notably younger, with higher BMI and systolic blood pressure, compared to patients who died from renal failure. This discovery could be a crucial step in preventing deaths from cerebro-cardiovascular disease among hemodialysis patients at core hospitals in rural areas of Japan. It underscores the importance of managing lifestyle habits to maintain a BMI of around 22, in addition to adequate blood pressure management.

## References

[1] Shibata S, Uchida S (2022). Hyperkalemia in patients undergoing hemodialysis: Its pathophysiology and management. Ther Apher Dial.

[2] Kobayashi S, Ohtake T (2023). The characteristics of dialysis membranes: benefits of the AN69 membrane in hemodialysis patients. J Clin Med.

[3] Zoccali C, Mallamaci F, Adamczak M (2023). Cardiovascular complications in chronic kidney disease: a review from the European Renal and Cardiovascular Medicine Working Group of the European Renal Association. Cardiovasc Res.

[4] Iguidbashian J, Imran R, Yi JA (2023). Maintenance and salvage of hemodialysis access. Surg Clin North Am.

[5] Tobe A, Sawano M, Kohsaka S (2023). Ischemic and bleeding outcomes in patients who underwent percutaneous coronary intervention with chronic kidney disease or dialysis (from a Japanese Nationwide Registry). Am J Cardiol.

[6] Hirayama A, Akazaki S, Nagano Y (2021). Hemodialysis raises oxidative stress through carbon-centered radicals despite improved biocompatibility. J Clin Biochem Nutr.

[7] Niikura R, Aoki T, Kojima T (2021). Natural history of upper and lower gastrointestinal bleeding in hemodialysis patients: a dual-center long-term cohort study. J Gastroenterol Hepatol.

[8] Kitajima A, Kishi T, Yamanouchi K (2023). A retrospective analysis of risk factors for mortality during hemodialysis at a general hospital that treats comprehensive diseases. Intern Med.

[9] Fujimoto S, Tsuruoka N, Esaki M (2021). Decline incidence in upper gastrointestinal bleeding in several recent years: data of the Japan claims database of 13 million accumulated patients. J Clin Biochem Nutr.

[10] Yoshikawa K, Kishi T, Takamori A (2024). Lower body bone fractures have high mortality rates and poor prognosis in the patients with hemodialysis. Ther Apher Dial.

[11] Kishi T, Kitajima A, Yamanouchi K (2022). Low body mass index without malnutrition is an independent risk factor for major cardiovascular events in patients with hemodialysis. Int Heart J.

[12] Nakayama S, Yamanouchi K, Takamori A (2024). Gastrointestinal bleeding among 151 patients undergoing maintenance hemodialysis for end-stage renal failure: a 5-year follow-up study. Medicine (Baltimore).

[13] Takagi K, Kishi T, Goto T (2024). Female patients with end-stage renal failure treated by hemodialysis had a low mortality rate and small patient number compared to male patients: 5-year follow-up study in Japan. J Clin Biochem Nutr.

[14] Ladhani M, Craig JC, Irving M, Clayton PA, Wong G (2017). Obesity and the risk of cardiovascular and all-cause mortality in chronic kidney disease: a systematic review and meta-analysis. Nephrol Dial Transplant.

[15] Polinder-Bos HA, Diepen MV, Dekker FW, Hoogeveen EK, Franssen CF, Gansevoort RT, Gaillard CA (2018). Lower body mass index and mortality in older adults starting dialysis. Sci Rep.

[16] Stenvinkel P, Gillespie IA, Tunks J (2016). Inflammation modifies the paradoxical association between body mass index and mortality in hemodialysis patients. J Am Soc Nephrol.

[17] Qazi HA, Chen H, Zhu M (2018). Factors influencing dialysis withdrawal: a scoping review. BMC Nephrol.

[18] Stenvinkel P, Heimbürger O, Lindholm B (2004). Wasting, but not malnutrition, predicts cardiovascular mortality in end-stage renal disease. Nephrol Dial Transplant.

[19] Inagaki K, Tawada N, Takanashi M, Akahori T (2022). The association between body mass index and all-cause mortality in Japanese patients with incident hemodialysis. PLoS One.

[20] Bhandari SK, Zhou H, Shaw SF (2022). Causes of death in end-stage kidney disease: comparison between the United States Renal Data System and a Large Integrated Health Care System. Am J Nephrol.

[21] Hecking M, Bieber BA, Ethier J (2014). Sex-specific differences in hemodialysis prevalence and practices and the male-to-female mortality rate: the Dialysis Outcomes and Practice Patterns Study (DOPPS). PLoS Med.

[22] Nitta K, Hanafusa N, Tsuchiya K (2017). Frailty and mortality among dialysis patients. Ren Replace Ther.

[23] Oki R, Hamasaki Y, Tsuji S (2022). Clinical frailty assessment might be associated with mortality in incident dialysis patients. Sci Rep.

[24] Wakasugi M, Narita I (2022). Sex differences in cause-specific mortality in Japanese dialysis patients. Intern Med.

[25] Joki N, Hase H, Nakamura R, Yamaguchi T (1997). Onset of coronary artery disease prior to initiation of haemodialysis in patients with end-stage renal disease. Nephrol Dial Transplant.

[26] Tanaka K, Watanabe T, Takeuchi A (2017). Cardiovascular events and death in Japanese patients with chronic kidney disease. Kidney Int.

[27] Denker M, Boyle S, Anderson AH (2015). Chronic renal insufficiency cohort study (CRIC): overview and summary of selected findings. Clin J Am Soc Nephrol.

